# Hypoxia enhances ILC3 responses through HIF-1α-dependent mechanism

**DOI:** 10.1038/s41385-020-00371-6

**Published:** 2021-01-14

**Authors:** J. L. Fachi, L. P. Pral, J. A. C. dos Santos, A. C. Codo, S. de Oliveira, J. S. Felipe, F. F. F. Zambom, N. O. S. Câmara, P. M. M. M. Vieira, M. Colonna, M. A. R. Vinolo

**Affiliations:** 1 Laboratory of Immunoinflammation, Department of Genetics, Evolution, Microbiology and Immunology, Institute of Biology, University of Campinas, Campinas, Brazil; 2 Department of Pathology and Immunology, Washington University School of Medicine, Saint Louis, MO USA; 3 Laboratory of Immunometabolism, Department of Genetics and Evolution, Microbiology and Immunology, Institute of Biology, University of Campinas, Campinas, Brazil; 4 Renal Division, Department of Clinical Medicine, Faculty of Medicine, University of São Paulo, São Paulo, Brazil; 5 Department of Immunology, Institute of Biomedical Sciences, University of São Paulo, São Paulo, Brazil; 6 Experimental Medicine Research Cluster, Campinas, Brazil; 7 Obesity and Comorbolities Research Center (OCRC), University of Campinas, Campinas, Brazil

## Abstract

Group 3 innate lymphoid cells (ILC3) have a prominent role in the maintenance of intestine mucosa homeostasis. The hypoxia-inducible factor (HIF) is an important modulator of immune cell activation and a key mechanism for cellular adaptation to oxygen deprivation. However, its role on ILC3 is not well known. In this study, we investigated how a hypoxic environment modulates ILC3 response and the subsequent participation of HIF-1 signaling in this process. We found increased proliferation and activation of intestinal ILC3 at low oxygen levels, a response that was phenocopied when HIF-1α was chemically stabilized and was reversed when HIF-1 was blocked. The increased activation of ILC3 relied on a HIF-1α-dependent transcriptional program, but not on mTOR-signaling or a switch to glycolysis. HIF-1α deficiency in RORyt compartment resulted in impaired IL-17 and IL-22 production by ILC3 in vivo, which reflected in a lower expression of their target genes in the intestinal epithelium and an increased susceptibility to *Clostridiodes difficile* infection. Taken together, our results show that HIF-1α activation in intestinal ILC3 is relevant for their functions in steady state and infectious conditions.

## Introduction

Oxygen constitutes ~21% of the atmosphere and it is commonly used as substrate for several biochemical reactions.^1^ Oxygen is the final electron acceptor in the mitochondrial electron transport chain, which is essential to convert nutrients into intracellular energy and to produce new biomolecules.^2^ However, oxygen can also be converted to several toxic free radicals that can induce modifications in proteins and DNA, damage to cellular organelles and even induce cell death.^1,2^ Hence, it is not a surprise that our cells present a variety of oxygen-sensing mechanisms for regulating its concentrations (i.e., capture, transport, and consumption) and adapt their metabolism and function to fluctuations in O_2_ levels.^3^

In the gastrointestinal (GI) tract, the oxygen tensions change across the length and longitudinally from the tissue in direction to the lumen, in which low levels of oxygen are observed at steady state.^4,5^ Intestinal epithelial cells (IEC) are adapted to this “physiological hypoxia”, which is essential for the proper function of the epithelium.^5^ Intestinal low oxygen availability activates responsive mechanisms in these cells, leading to the expression of genes crucial to the mucosal barrier functionality, such as junctional proteins, antimicrobial peptides, and mucus.^6,7^ Thereby, other cells present in the intestinal mucosa also have to adapt their metabolism and functions according to O_2_ fluctuations in the GI tract.

One of the main mechanisms involved on the cellular adaptation to hypoxic environments is hypoxia-inducible factor (HIF)-1 activation. Expressed in practically all cells of the immune system, as well as in IECs, HIF-1 is a transcription factor composed of 2 subunits, a constitutive subunit called HIF-β and HIF-1α. The stability of HIF-1α is regulated through different mechanisms, including at the post-translational level by prolyl-hydroxylases (PHDs).^8^ At normal O_2_ levels, iron-dependent PHDs are activated and hydroxylate HIF-1α, which leads to proteasomal ubiquitination mediated by von Hippel-Lindau tumor suppressor protein.^8,9^ By contrast, in low O_2_ levels, PHDs are inactivated, resulting in intracellular accumulation of HIF-1α, which together with HIF-β and the co-activator p300, translocate to the nucleus, bind to hypoxia-responsive elements, and thus regulate the expression of several genes related to cellular adaptation in the hypoxic environment.^10–12^

Group 3 innate lymphoid cells (ILC3), which are abundant in the intestinal mucosa, are characterized by expression of the transcription factor RORγt and cytokines such as IL-17, IL-22, and GM-CSF.^13^ These cells are known to respond to signals such as IL-1β and IL-23 released by resident cells following tissue damage^14^ and to participate in regulation of the intestinal barrier and host-microbiota interactions.^15^ ILC3 also contribute to the response during intestinal infectious conditions, such as those caused by *Citrobacter rodentium* and *Clostridioides difficile.*^16–18^

In this study, we explored the role of hypoxia and HIF-1 in the ILC3 responses. We demonstrate that hypoxia increased numbers, proliferation and activation of ILC3 through a HIF-1α-dependent mechanism. Using conditional knockout mice and pharmacological inhibitor, we found that hypoxia-induced ILC3 activation is due to a direct ability of HIF-1α to modulate the transcriptional state, via expression of RORγt and target-genes. This process is independent of HIF-1α-induced metabolic regulation. Finally, our study indicates that in the absence of HIF-1α signaling, mice are more susceptible to intestinal infection by *C. difficile*. These results reveal an important role of hypoxia and HIF-1 stabilization in ILC3, which functionally modulate their transcriptional activation and performance in the intestinal lamina propria.

## Results

### Low oxygen levels are associated with higher ILC3 response

**Fig. 1:**
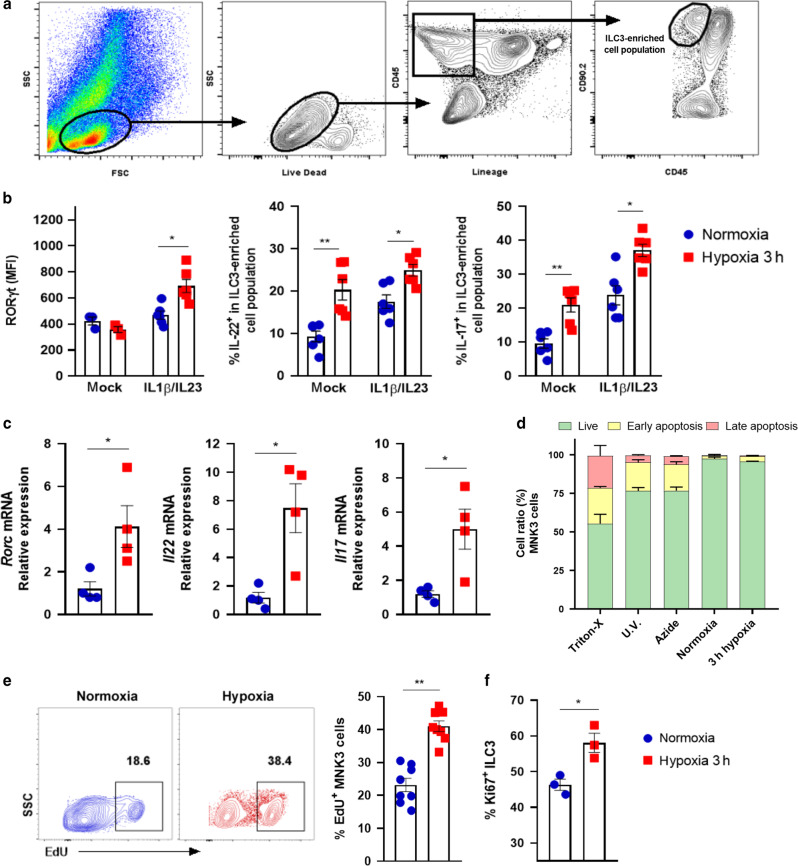
Hypoxia increases ILC3 activation and proliferation. **A** Gating strategy of live Lin^-^CD45^low^CD90.2^high^ enriched ILC3 population from small intestine (si) lamina propria (as shown in Fig. S1). **B** RORγt mean fluorescence intensity and production of IL-17 and IL-22 by enriched si-ILC3 subset obtained after incubation for 3 h, under normoxia/hypoxia. Cells were non-stimulated (mock) or stimulated with IL-1β + IL-23 ex vivo (*n* = 6). **C** Relative *Rorc*, *Il22* and *Il17* mRNA expression in stimulated MNK3 cells under normoxia/hypoxia (*n* = 4). **D** Cellular viability of MNK3 cells incubated 3 h in hypoxia/normoxia using annexin-V/7-AAD staining. Live (green) represents the double-negative population, early apoptosis (yellow) shows annexin-V^+^7-AAD^−^, and late apoptosis (red) is double positive cells (*n* = 4). Percentage of EdU^+^ MNK3 cells (**E**) and Ki67^+^ cells in the ILC3-enriched population (**F**) as measured by flow cytometry (*n* = 7 and 3, respectively). Representative EdU^+^ plots are presented on the left. Results are representative of at least two independent experiments and presented as mean ± SEM. *p < 0.05; **p < 0.01.

We first tested whether low levels of oxygen are associated with small intestine (si)-ILC3 activation. Cells were incubated for 3 h in hypoxia or normoxia in the presence or not of IL-1β/IL-23 stimuli and then were analyzed by flow cytometry using the gate strategy described in Fig. 1a and Supplementary Fig. S1.^19–21^ Under hypoxia, IL-1β/IL-23-stimulated Lin^-^CD45^low^CD90.2^+^ cells (ILC3-enriched cell population) had higher levels of RORγt, IL-22 and IL-17 proteins than normoxic cells (Fig.1b). In particular, IL-22 and IL-17 were modulated by hypoxia itself, regardless of the stimuli. These results were corroborated by an increase in relative gene expression in the MNK3 ILC3 cell line (Fig. 1c), indicating that low levels of oxygen were associated with greater ILC3 activation. The observed phenotypes were not associated with any effect on MNK3 cell viability, as demonstrated by Annexin-V/7-ADD staining (Fig. 1d). Likewise, in hypoxia, ILC3 proliferation was increased, as demonstrated by higher incorporation of EdU in MNK3 cells and expression of the proliferation marker Ki67 in primary ILC3s (Fig. 1e, f). For hypoxia induction, we used an anaerobic gas generator, which consumes all oxygen within 3 h, similar to Dang et al.^22^ The results were confirmed in MNK3 cells incubated for the same period with controlled oxygen levels (8% O_2_) and compared with cells in normoxia (Supplementary Fig. S2A and S2B).

### ILC3 activation in hypoxia is associated with HIF-1α stabilization

**Fig. 2:**
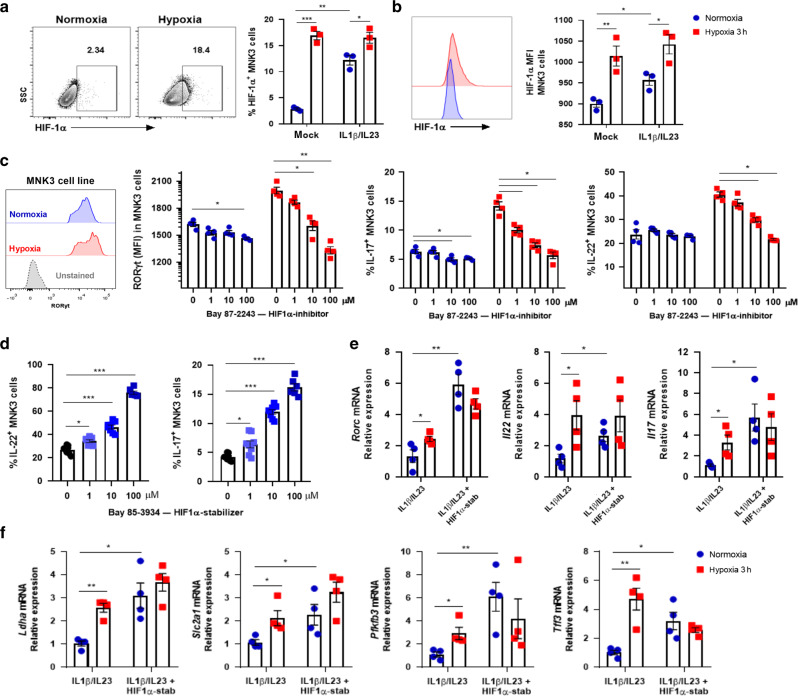
Hypoxia-induced HIF-1α stabilization boosts ILC3 cell line activation. HIF-1α content in MNK3 cells incubated for 3 h under hypoxia/normoxia, as measured by flow cytometry. Graphs indicate **A** percentage of HIF-1α^+^ cells and **B** HIF-1α geometric MFI (*n* = 3). Representative FACS plots on the left. RORγt geometric MFI and IL-17 and IL-22 content in stimulated MNK3 cells after 3 h in normoxia/hypoxia with different concentrations of HIF-1α inhibitor, Bay 87–2243 (inhib) (**C**), or stabilizer, Bay 85–3934 (stab) (**D**) (*n* = 4 and 6, respectively). A representative histogram of RORγt expression in MNK3 cells is shown in figure C on the left. Relative mRNA expression of *Rorc*, *Il22*, *Il17* (**E**), and HIF-1 target genes (*Ldha*, *Slc2a1*, *Pfkfb3,* and *Tff3*) (**F**) of MNK3 cells stimulated during 3 h in normoxia/hypoxia and treated or not with HIF-1α stabilizer (n = 4). Results are representative of at least two independent experiments and presented as mean ± SEM. *p < 0.05; **p < 0.01; ***p < 0.001.

To assess whether the hypoxia-induced effect was dependent on exposure time, cells were incubated for 1 h at hypoxia or normoxia. This time was enough to increase the expression of RORγt, IL-22, and IL-17 in MNK3 cells (Supplementary Fig. S2C). Considering the rapid response to oxygen fluctuation, we next evaluated stabilization of HIF-1α in the MNK3 cell line incubated under normoxia/hypoxia. We observed that cytokine-stimulated cells presented an increase in HIF-1α levels compared to unstimulated controls in normoxia (Fig. 2a, b). The levels of HIF-1α were increased in cells incubated in hypoxic atmosphere. This occurred regardless of IL-1β/IL-23 stimulation (Fig. 2a, b). Additionally, we analyzed by FACS the content of HIF-1α in ILC3 subsets. NCR^+^ and CCR6^+^ ILC3 subsets expressed comparable levels of HIF-1α, while NCR^-^ ILC3 showed a small increase in the content of this protein compared to the other subsets (Fig. S2D).

To gain insight into the relevance of HIF-1 in the ILC3 phenotype under low oxygen levels, we tested the effect of its pharmacological activation and inhibition. A potent and selective inhibitor of HIF-1α (Bay 87–2243) induced a dose-dependent reduction of MNK3 activation in normoxia and hypoxia (Fig. 2c). In contrast, the HIF-1α prolyl-hydroxylase inhibitor (Bay 85–3934), which stabilizes HIF-1α even in the presence of oxygen, showed the opposite phenotype in normoxia, as increased Bay 85–3934 concentrations led to increased production of IL-22 and IL-17 by MNK3 cells (Fig. 2d). Under low levels of O_2_, we did not observe any additional effect of HIF-1α stabilization following Bay 85–3934 treatment on expression of genes related to MNK3 activation (Fig. 2e). We further confirmed that HIF-1 was more active in MNK3 cells under hypoxia by incubating with Bay 85–3934 and evaluating the expression of its target genes *Ldha*, *Slc2a1*, *Pfkfb3*, and *Tff3*, which were all increased (Fig. 2f).

### Hypoxia-boosted ILC3 activation is independent of mTOR signaling

**Fig. 3:**
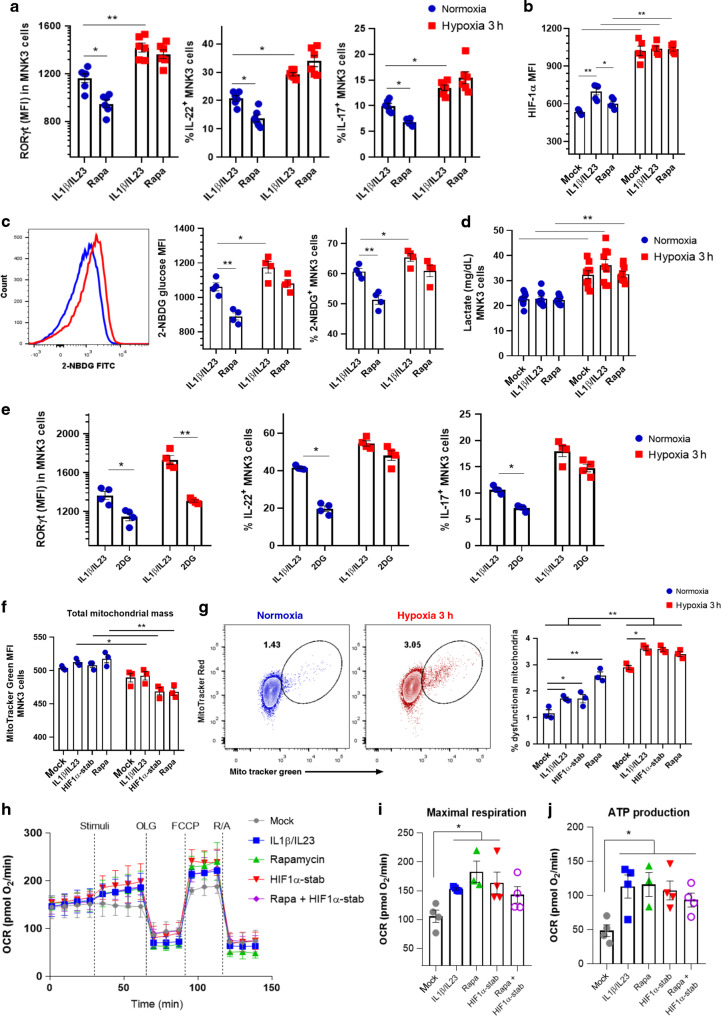
ILC3 cell line under hypoxia do not depend on glycolytic metabolism and mitochondrial respiration. **A** RORγt geometric MFI and IL-22 and IL-17 content in stimulated MNK3 cells after 3 h under normoxia/hypoxia with and without rapamycin treatment (*n* = 6). **B** HIF-1α geometric MFI in MNK3 cells incubated 3 h under normoxia or hypoxia with different conditions: unstimulated (mock); IL-1β and IL-23 stimulated; and IL-1β, IL-23 and rapamycin (Rapa) treated (*n* = 5). **C** Uptake of glucose (2-NBDG) by flow cytometry in stimulated MNK3 cells with and without rapamycin treatment (*n* = 4). A representative histogram is on the left and graphs showing MFI and percentages are on the right. **D** Lactate release by MNK3 cells during 3 h incubation in hypoxia/normoxia under different conditions, as described in B (*n* = 9). **E** RORγt geometric MFI and IL-22 and IL-17 content in stimulated MNK3 cells, treated with 2DG for 3 h under normoxia/hypoxia (*n* = 4). Total mitochondrial mass (**F**) and mitochondrial membrane potential (**G**) in MNK3 cells labeled with MitoTracker Green and MitoTracker Red after 3 h incubation in normoxia or hypoxia (*n* = 3). Cells were stimulated with IL-1β + IL-23 and treated or not treated with HIF-1α stabilizer (stab) or rapamycin. Real-time changes (**H**), maximal respiratory capacity (**I**), and ATP production (**J**) in the OCR of MNK3 cells after stimulation and treatment with oligomycin (OLF), FCCP, and rotenone/antimycin A (R/A) (*n* = 4). Cells were stimulated or not with IL-1β + IL-23 and treated or not with HIF-1α stabilizer (stab) and/or rapamycin. Results are performed at least twice (except H-J) and presented as mean ± SEM. *p < 0.05; **p < 0.01.

To gain insight into the mechanisms involved in the activation of ILC3 under hypoxia, we first tested whether ILC3 relies on mammalian target of rapamycin (mTOR) signaling for metabolic adaptation to low oxygen levels. The mTOR pathway is a central regulator of mammalian metabolism and physiology, acting as a protein kinase that regulates complex cellular functions.^23^ As already shown,^20^ rapamycin, a mTOR inhibitor, decreases MNK3 expression of RORγt, IL-22, and IL-17 in normoxia, which indicates that mTOR sustains cell-intrinsic activation (Fig. 3a). However, the increased expression of RORγt, IL-22, and IL-17 observed during hypoxia was not affected by the presence of rapamycin (Fig. 3a), indicating that ILC3 cell line activation at low oxygen levels is independent of mTOR signaling. Since mTORC1 drives glycolytic metabolism and mTOR is related to HIF-1α stabilization, we next measured HIF-1α expression in MNK3 cells incubated with rapamycin in normoxia and hypoxia. Rapamycin reduced HIF-1α expression in stimulated MNK3 in normoxia, an effect that was absent in cells incubated in hypoxia (Fig. 3b). Similarly, IL-23/IL-1β-activated MNK3 cells under hypoxia had increased glucose uptake and produced more lactate than cells in normoxia (Fig. 3c, d). Although rapamycin reduced glucose uptake and lactate production in normoxic atmosphere, no effects were observed under hypoxic conditions, which further indicates that ILC3 do not depend on mTOR signaling at low oxygen levels (Fig. 3c, d). Contributing to the hypothesis that hypoxia-boosted ILC3 activation is independent of glycolytic metabolism, inhibition of glycolysis with 2-DG had only a minor effect in MNK3 acute response under hypoxia. Despite the RORγt expression, the IL-17 and IL-22 levels were still elevated after 2-DG treatment in hypoxia compared to normoxia (Fig. 3e). Together, these data suggest that boosted activation of ILC3 by hypoxia was independent of glycolysis and mTOR activation.

### Hypoxia boosts ILC3 activation independently of mitochondrial fitness

To determine whether oxygen levels modify the cellular energy program, since we investigated the total mitochondrial content of MNK3 cells with MitoTracker Green. Different combinations of IL-23, IL-1β, rapamycin, and HIF-1α stabilizer did not affect mitochondrial mass in normoxia and reduced mitochondrial mass under hypoxia. This effect was more evident when cells were treated with rapamycin or HIF-1α stabilizer (Fig. 3f). We also evaluated the mitochondria function, distinguishing between mitochondria with (respiring) and without (dysfunctional) membrane potential. We observed increased dysfunctional mitochondria load (MitoTracker Red^+^MitoTrackerGreen^high^) in stimulated-MNK3 cells treated with rapamycin and HIF-1α stabilizer under normoxia (Fig. 3g). In addition, low oxygen levels potentiated mitochondria dysfunction, even when the mTOR pathway was inhibited. Next, we assessed the metabolic profile of cells with blocked mTORC activity (rapamycin) and increased HIF-1 levels (stabilizer) by real-time analysis of oxygen consumption rate (OCR) at normal oxygen level (Fig. 3h). Unstimulated MNK3 cells had lower maximal respiratory capacity and ATP production than cells stimulated with IL-1β/IL-23 (Fig. 3h j), suggesting increased energy demand. However, no difference on OCR was observed in MNK3 cells treated with rapamycin, HIF-1α stabilizer or combination of both, suggesting a mTORC-independent effect of HIF-1 on ILC3 cell line response. We also observed no differences on basal respiration, non-mitochondrial oxygen consumption, spare respiratory capacity, and acute response by the OCR analysis (Supplementary Fig. S3A–D). The energy map showed higher overall energetic profile instead of a glycolytic dominant profile in stimulated MNK3 compared to resting cells (Supplementary Fig. S3E). Rapamycin and HIF-1α increased levels did not show major differences compared to stimulated cells (Supplementary Fig. S3E). These data indicate that hypoxia boosts ILC3/MNK3 activation through a HIF-1-dependent mechanism and independently of glycolysis and mitochondrial fitness.

### The transcriptional interaction of HIF-1α-RORγt is crucial for hypoxia-activated ILC3

**Fig. 4:**
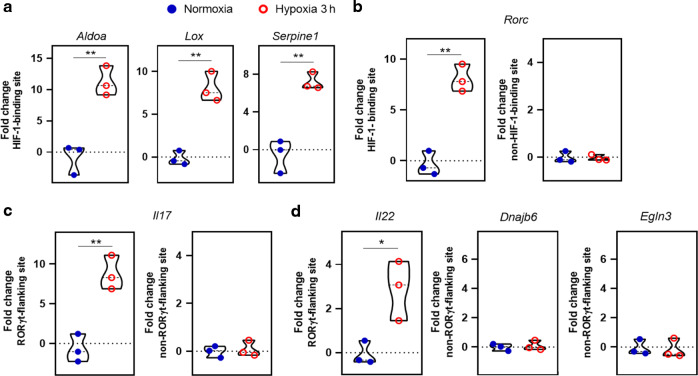
Hypoxia induces HIF-1α-RORγt interaction and transcriptional fate in ILC3. Analysis of HIF-1 interaction with genes using the chromatin immunoprecipitation (ChIP) technique. MNK3 cells were incubated for 3 h in hypoxia or normoxia without stimulation and then HIF-1α-associated chromatin sites were purified and amplified by qPCR (*n* = 3). **A** Measurement of HIF-1α binding to HIF-1α-responsive genes under hypoxia. **B** Measurement of HIF-1α binding to known HIF-1-binding or non-binding sites of *Rorc*. **C** Measurement of HIF-1α binding to the RORγt-flanked or not flanked sites of *Il17*. **D** Measurement of HIF-1α binding to *Il22* and non-RORγt-flanking *Dnajb6* and *Egln3* genes. All ChIP-qPCR data were normalized by log_2_-fold change of total chromatin extraction (input) to purified immunoprecipitated chromatin. Results are representative of two independent experiments and presented as mean ± SEM. *p < 0.05; **p < 0.01.

Chromatin immunoprecipitation (ChIP) assay was performed for identifying genomic loci that directly or indirectly interact with HIF-1α in MNK3 cells. As expected, we identified known HIF-1 target genes that directly bound to HIF-1α and were upregulated in hypoxia, such as aldolase (*Aldoa*), lysyl oxidase (*Lox*), and *Serpine1* (Fig. 4a). RORγt (*Rorc*) gene also showed approximately eightfold higher interaction with HIF-1α in hypoxia than normoxia, suggesting that HIF-1 directly regulates *Rorc* expression (Fig. 4b). Similarly, immunoprecipitation was enriched for genes associated with ILC3 function, *Il17* and *Il22*, through the RORγt-flanking site (Fig. 4c, d), indicating that ILC3-related genes are transcriptionally regulated by HIF-1α under hypoxia.

### HIF-1α deletion results in reduced numbers and less functional ILC3

**Fig. 5:**
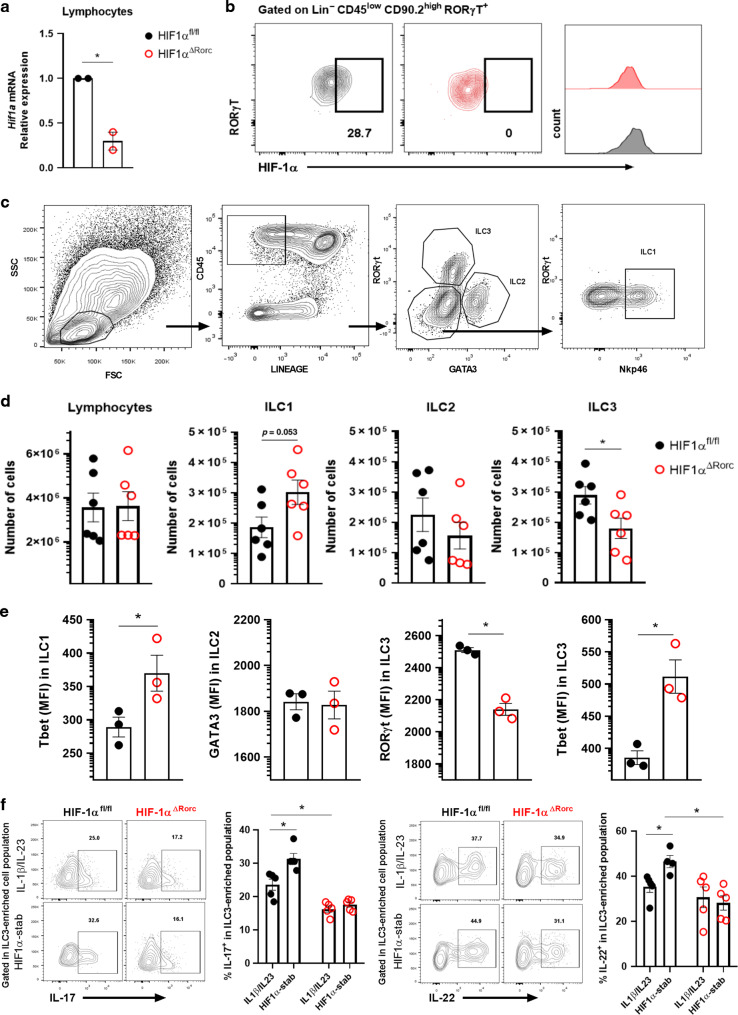
HIF-1α^ΔRorc^ mice have lower number and less functional ILC3 at steady state. Relative *Hif1a* mRNA expression in total lymphocytes (**A**) and FACS plots of HIF-1α content in Lin^-^CD45^low^CD90.2^high^RORγt^+^ ILC3 (**B**) from HIF-1α^ΔRorc^ and HIF-1α^floxed^ small intestine lamina propria. Cells were incubated for 2 h in hypoxia (n = 2). **C** Gating strategy to identify and quantify ILC subsets in intestinal lamina propria. **D** Absolute number of ILC subsets in HIF-1α^ΔRorc^ and HIF-1α^floxed^ small intestine lamina propria (*n* = 5–6). ILC1, ILC2 and ILC3 are defined as Lin^-^CD45^+^RORγt^-^Gata3^-^Nkp46^+^, Lin^-^CD45^+^RORγt^-^Gata3^+^ and Lin^-^CD45^+^RORγt^+^Gata3^-^, respectively. Lineage was defined by CD3, CD5, CD19, CD11b, and CD11c markers. **E** Geometric mean fluorescence intensity of T-bet in ILC1, Gata3 in ILC2, and T-bet and RORγt in ILC3 from small intestine lamina propria of HIF-1α^ΔRorc^ and HIF-1α^floxed^ mice (*n* = 3). **F** Percentage of IL-17 and IL-22 producing Lin^-^CD45^low^CD90.2^+^ (ILC3-enriched population) cells from HIF-1α^ΔRorc^ and HIF-1α^floxed^ mice. Representative FACS plots are presented on the left. Cells were stimulated with IL-1β + IL-23 ex vivo and treated or not with the HIF-1α stabilizer (stab) (*n* = 5). All mice were littermates and matched by sex and age. Results are representative of at least two independent experiments and presented as mean ± SEM. *p < 0.05.

Next, we used HIF-1α conditional knockout mice to evaluate the physiological role of this hypoxia-induced transcription factor on ILC3 in RORγt expressing cells (Fig. 5a, b). HIF-1α deficiency in RORγt^+^ cells (KO), which included ILC3, was associated with a significant reduction of si-ILC3 number (Fig. 5c, d), and lower expression of RORγt and IL-22/IL-17 in ILC3 compared to the control littermates (HIF-1α^floxed^ mice, or WT) (Fig. 5e, f). This effect was accompanied by an increase of ILC1 numbers and its T-bet expression and no effect on ILC2 (Fig. 5d, e). ILC3-enriched cell population from HIF-1α deficient mice, in opposition to cells from HIF-1α sufficient mice, were not responsive to HIF-1α stabilizer treatment ex vivo, confirming the hypothesis that HIF-1 is relevant for the effect of this drug on ILC3 (Fig. 5f). No differences were observed in expression of *Il23r* or *Il1r* in MNK3 cells after 3 h in hypoxia compared to normoxia (Supplementary Fig. S4A) or in HIF-1α-deficient ILC3 compared to wild-type cells (Supplementary Fig. S4B), indicating that hypoxia-induced HIF-1α stabilization does not improve ILC3 responsiveness to IL-23 and IL-1β. Likewise, knocking out HIF-1α in IEC did not result in quantitative or functional differences in ILCs, particularly ILC3 (Supplementary Fig. S4C–G).

**Fig. 6:**
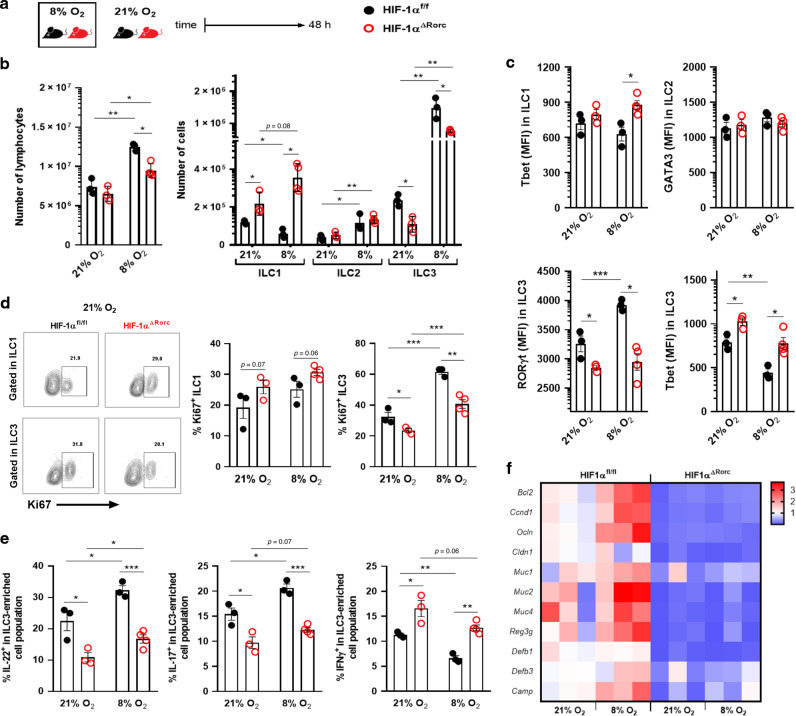
Acute hypoxia exposure delays ILC3 responses in a HIF-1 manner. **A** Experimental scheme of HIF-1α^ΔRorc^ and HIF-1α^floxed^ mice housed under normoxia or 8% O_2_-hypoxia, using a normobaric gas chamber, for 48 h. **B** Absolute number of ILC subtypes in small intestine lamina propria (*n* = 3–4). ILC1, ILC2, and ILC3 are defined as Lin^-^CD45^+^RORγt^-^Gata3^-^Nkp46^+^, Lin^−^CD45^+^RORγt^−^Gata3^+^ and Lin^−^CD45^+^RORγt^+^Gata3^-^, respectively. **C** Geometric MFI of T-bet in ILC1, Gata3 in ILC2, T-bet and RORyt in ILC3 from si-lamina propria of HIF-1α^ΔRorc^ and HIF-1α^floxed^ mice kept in normoxia or hypoxia (*n* = 3–4). **D** Percentage of Ki67^+^ ILC1 (Lin^-^Tbet^+^, left) and ILC3 (Lin^-^RORγt^+^, right) under normoxia or hypoxia (*n* = 3–4). Representative FACS plots are presented on the left. **E** Percentage of IL-22, IL-17, and IFN-γ producing Lin^-^CD45^low^CD90.2^+^ (si-ILC3-enriched population) cells from HIF-1α^ΔRorc^ and HIF-1α^floxed^ mice in normoxia or hypoxia (*n* = 3–4). **F** Relative IL-22-target gene expression in distal ileum under normoxia or hypoxia (*n* = 4). All HIF-1α^ΔRorc^ and HIF-1α^floxed^ mice were littermates and matched by sex and age. Results are presented as mean ± SEM. *p < 0.05; **p < 0.01; ***p < 0.001.

Next, we tested whether mice subjected to a hypoxic condition would show differences in ILC3s activation and some of their targets in the small intestine. HIF-1α deficient mice (HIF-1α^ΔRorc^ or KO) and littermate controls (HIF-1α^fl/fl^ or WT) were maintained in a normobaric chamber at 8% oxygen for 48 h and compared with mice in normoxia (Fig. 6a). Wild-type mice showed an increase of lymphocytes, ILC2 and ILC3 number, as well as reduction of ILC1, in the SI when maintained under hypoxia and compared to normoxia controls (Fig. 6b). HIF-1α^ΔRorc^ mice also presented an increased number of lymphocytes, ILC2 and, although less pronounced, ILC3 in hypoxia compared to normoxia (Fig. 6b). Comparing KO to WT mice, the HIF-1α absence was associated to reduced ILC3 and increased ILC1 frequency, regardless of oxygen level (Fig. 6b). This phenotype was accompanied by lower RORγt and increased T-bet expression in ILC3 compartment from KO mice (Fig. 6c). No effect was observed on the ILC2 expression of Gata3 (Fig. 6c). We also observed increased proliferation of ILC3-enriched population in WT mice under hypoxia, which was reduced in KO, and greater tendency of ILC1 to proliferate in KO mice in both normoxia or hypoxia (Fig. 6d), suggesting a shift on the ILC1/ILC3 ratio at lower O_2_ levels or HIF-1α deficiency. We also found higher expression of IL-22, IL-17, and their target genes in the small intestine of WT mice in hypoxia compared to normoxia (Fig. 6e, f). These effects were attenuated when HIF-1α was deleted (Fig. 6e, f).

### HIF-1α-deficient Rorc mice are more susceptible to C. difficile infection

**Fig. 7:**
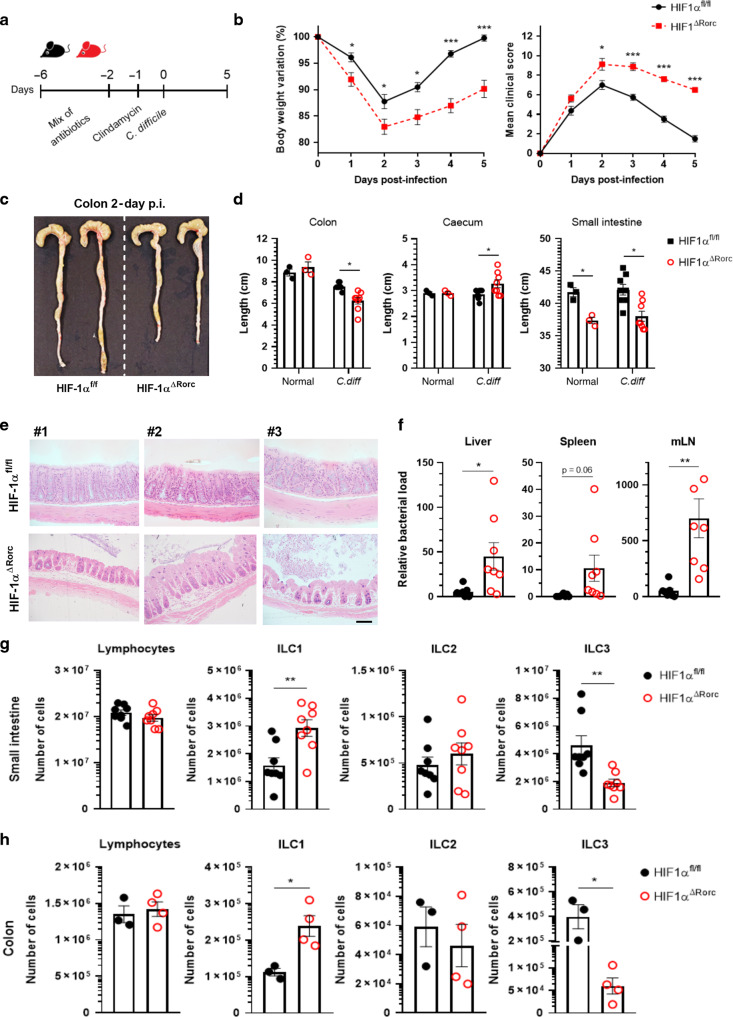
HIF-1α^ΔRorc^ mice are more susceptible to *C. difficile* infection. **A** Scheme of *C. difficile* infection model. HIF-1α^ΔRorc^ and HIF-1α^floxed^ received a mixture of antibiotics for 4 days and then received a single i.p. dose of clindamycin. Mice were then infected with 10^8^ CFU of *C. difficile*. **B** Body weight changes and clinical score during *C. difficile* infection (*n* = 8). Image of colons (**C**) and lengths (**D**) of colon, cecum and small intestine from *C. difficile*-infected mice on day 5 (*n* = 3–8). **E** Representative histological sections of colons stained with hematoxylin and eosin from *C. difficile-*infected mice on day 5 (*n* = 4). Scale bar represents 100 μm. **F** Bacterial translocation into the mesenteric lymph nodes (mLN), spleen and liver assessed by qPCR at day 5 (*n* = 7–8). Absolute number of Lin^-^CD45^+^ ILC subtypes in small intestine (**G**) or colon (**H**) lamina propria from infected mice on day 5 (*n* = 3–8). All HIF-1α^ΔRorc^ and HIF-1α^floxed^ mice were littermates and matched by sex and age. Results are pools of two independent experiments and presented as mean ± SEM. *p < 0.05.

**Fig. 8:**
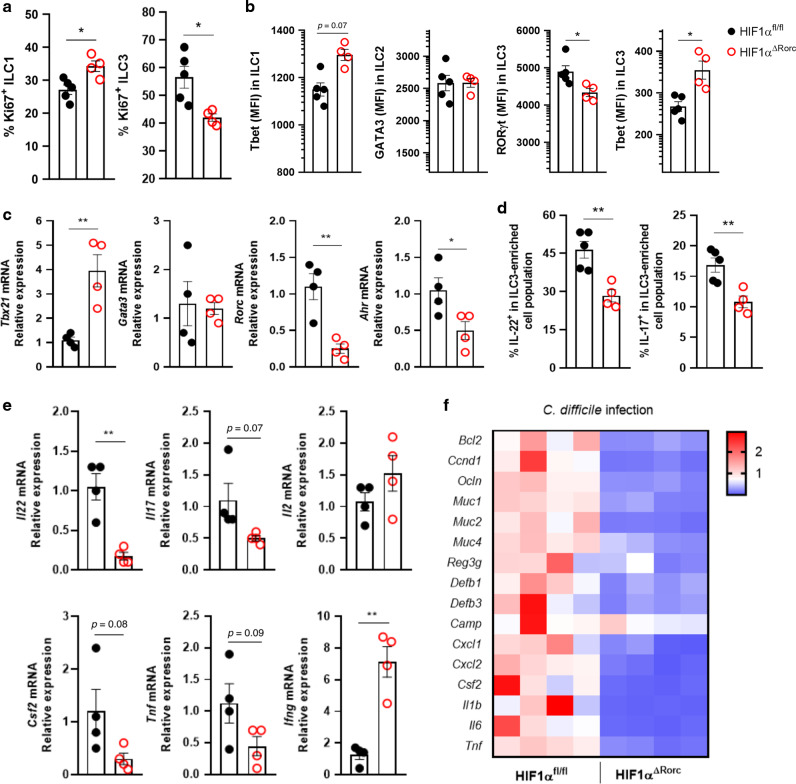
HIF-1α^ΔRorc^ infected mice have impaired ILC3. **A** Percentage of Ki67^+^ ILC1 (left) and ILC3 (right) from HIF-1α^floxed^ and HIF-1α^ΔRorc^ infected mice on day 5 (*n* = 4–5). **B** Geometric MFI of T-bet expression in ILC1, Gata3 in ILC2, and T-bet and RORγt in ILC3 from small intestine lamina propria of *C. difficile* infected mice on day 5 p.i. (*n* = 4–5). **C** Relative *Tbx21*, *Gata3*, *Rorc*, *Ahr* mRNA expression in total lymphocytes from small intestine lamina propria of infected mice on day 5 (*n* = 4). **D** IL-22 and IL-17 producing Lin^-^CD45^low^CD90.2^+^ (si-ILC3-enriched population) cells from infected HIF-1α^ΔRorc^ and HIF-1α^floxed^ mice on day 5 p.i. Cells were stimulated ex vivo with IL-1β and IL-23 for 3 h (*n* = 4). **E** Relative cytokine mRNA expression in total lymphocytes from small intestine lamina propria of infected mice on day 5 p.i. (*n* = 4). **F** Relative IL-22 and IL-17-target gene expression in the proximal colon of HIF-1α^ΔRorc^ and HIF-1α^floxed^ mice on day 5 p.i. (*n* = 4). All HIF-1α^ΔRorc^ and HIF-1α^floxed^ mice were littermates and matched by sex and age. Results are representative of two independent experiments and presented as mean ± SEM. *p < 0.05; **p < 0.01.

To determine the relevance of HIF-1α in the role of ILC3 during an inflammatory/infectious condition, we used the *C. difficile* infection (CDI) model, previously shown to have an important role of ILC3 and IL-22.^17,21,24^ We infected HIF-1α conditional knockout mice with *C. difficil*e and assessed the impact of HIF-1α on ILC3 function during colitis (Fig. 7a). We observed increased susceptibility of KO to CDI compared to WT. KO mice presented higher clinical scores, lost more body weight during infection, and displayed significant reduced size of the colon and small intestine compared to WT (Fig. 7b–d). We also observed high tissue damage and increased translocated bacterial to the liver, spleen and mesenteric lymph nodes in KO mice, consistent with the reduction in intestinal barrier function and immune response during the acute phase of infection (Fig.7e, f). Similar to uninfected mice (Fig. 5), we observed a significant reduction in the number of ILC3 and increased ILC1 in the colon and SI of KO compared to WT mice (Fig. 7g, h). HIF-1α deficient mice also presented several functional changes in the ILC1/ILC3 ratio (Fig. 8). Increased ILC1 proliferation, T-bet content and *Ifng* mRNA expression was observed in infected KO compared to WT mice (Fig. 8a–e). On the other hand, a reduction of ILC3 proliferation and activation was observed in the absence of HIF-1α in the RORγt^+^ compartment (Fig. 8a–e). These findings were accompanied by a downregulation of IL-22 and IL-17 target genes expression in the colon of infected HIF-1α-deficient mice (Fig. 8f), suggesting that ILC3 activation by HIF-1 signaling has a crucial role in maintaining the intestinal mucosal barrier and is a key component for mucosal immune response during intestinal *C. difficile* infection (Supplementary Fig. S5).

## Discussion

We demonstrated that low oxygen levels boost intestinal ILC3 activation and proliferation. The observed phenotype was lost by deleting or chemically inhibiting HIF-1α stabilization, showing that functional ILC3 responses to hypoxia lean towards HIF-1 signaling. Previous studies have shown participation of HIF-1 in functional control of different immune cell subsets.^25^ Hypoxia-induced HIF-1 activation improves differentiation, proliferation and metabolic reprogramming of Th17 and Treg cells.^22,26–28^ The antitumor capacity of CD8 T cells is also increased by HIF-1 via induction of glycolytic metabolism, tumor infiltration and tumoricidal memory formation.^29,30^ Likewise, recent studies have shown participation of HIF-1 in the innate lymphoid compartment. Tumor-infiltrated NK cells operate under hypoxia and rely on HIF-1 to cytokine production and tumor cell death induction.^31^ HIF-1 activation also directly affects late ILC2 maturation through the IL33-ST2 signaling pathway.^32^ Together, our results and published reports highlight the relevance of the HIF-1 pathway for steady state immune system functions in intestinal mucosa and lymphoid tissue, and in tumoral, ischemic and inflamed environments.

Massive cellular activities require energy-generating precursors for lipid, nucleotide and amino acid synthesis, necessary for an effective response, such as proliferation and gene expression. Naive T cells use oxidative phosphorylation as the main source of energy, different from activated T cells, that shifts to aerobic glycolysis and glutamine catabolism for energy production and biosynthetic precursors.^33,34^ Th17 development is improved by the mTOR-HIF-1α axis, which controls transcriptional activation of RORγt and sustains glycolysis.^22,28^ The mTORC1-HIF-1α axis also sustains ILC3 responses toward glycolysis and RORγt expression in normoxia.^20^ Although ILC3 and Th17 overlap, ILC3 activation increases mitochondrial respiration and its production of reactive oxygen species (mROS), that stabilizes HIF-1α under normoxia and inhibits pyruvate dehydrogenase kinase, in contrast to Th17.^20,35^ However, our work showed that activation of ILC3 in hypoxia does not depend on glycolysis or mitochondrial respiration. Similarly, Velasquez *et al*. showed that IL-15-stimulated NK cells were more activated under acute hypoxia, with increased glycolytic gene expression without major changes in glycolytic flux or glucose consumption.^36^ In addition, HIF-1 disrupts the pattern of genes expressed by cells enabling it to interact with other transcription factors and regulate their stability or activity. This has already been demonstrated for RORγt and Foxp3 in T helper cells.^22^ Thus, here we showed that HIF-1 activation in hypoxia boosts ILC3 activation through its transcriptional ability to increase the RORγt expression and activity. Further investigation will be necessary to validate the molecular HIF-1-RORγt interaction.

We have also observed a crucial role in hypoxia-induced HIF-1α activation for maintaining intestinal mucosa and increasing resistance to *C. difficile* infection. Healthy intestinal microbiota brings mutual benefits and assists in resistance to colonization by pathogens. However, due to the use of antibiotics and microbiota imbalance, *C. difficile* proliferates and produces toxins, causing inflammation in colon mucosa, which ranges from benign to fulminant colitis.^37^ Microbiota contributes to low oxygen content in the intestinal lumen, which is relevant for its host interactions along the whole intestinal tract.^38,39^ HIF-1α stabilization in IECs functionally ensures barrier integrity and colitis protection.^6,7^ Colitis-induced intestinal epithelium disruption causes exposure of luminal bacteria and excessive innate inflammatory responses, related to tumor or lethal colitis development.^40^ We found that intestinal oxygen levels can act on ILC3 activation, inducing increased ILC3 target-gene expression in the epithelium, which is important to maintain the barrier homeostasis. Deletion of HIF-1 in ILC3s compartment impaired the epithelial barrier and the mechanisms controlling bacterial translocation, showing relevance of HIF-1 in the ILC3 role. A previous study showed that deletion of HIF-1 in T cells also affected host protection in DSS-induced colitis and increased cellular infiltration,^41^ highlighting the relevance of HIF-1 expression and stabilization in immune cell types, and the epithelium.

Likewise, we found that mice exposed to acute hypoxia at 8% oxygen had a significant functional influence on activation of intestinal ILC3 through subsequent HIF-1 signaling. Low oxygen levels boosted ILC3 activation and cytokine release, impacting on tissue gene expression and mucosal homeostasis. This may have important implications for individuals regularly exposed to drastic changes in the oxygen pressures, such as long-haul flights and differences in sea level (21% O_2_) and mountain locations, where oxygen availability drops dramatically (~11% O_2_).^11^ We also observed an interesting effect of hypoxia and HIF-1 on the ILC1/ILC3 balance in the intestine. As ILC3 expresses RORγt and resembles Th17 cells, ILC1 (including NK cells) expresses the transcription factor T-bet and secretes mainly IFN-γ, similar to Th1 responses.^13^ We found that hypoxia-induced HIF-1 signaling increases RORγt expression and IL-22/IL-17 production and reduces T-bet and IFN-y. Consistently, we noticed greater accumulation of ILC1 in the lamina propria, and enhanced expression of T-bet and IFN-y in HIF-1α knockout mice. Recent work indicates the presence of several subsets of ILC3 and high plasticity between these populations and other populations of ILCs.^42^ Emerging RORγt + ILC3 acquires T-bet expression for subsequent expression of NRC, showing an ILC1-like phenotype and production of TNF, IFN-γ, and IL-22 with lower IL-17.^43–45^ Thus, we speculate that HIF-1α may guarantee the intact RORγt-ILC3 profile and minimize their plastic capacity to the ILC1-like phenotype. This aspect and others, such as the role of hypoxia and HIF-1 in the intestinal lymphoid tissue formation, their effects on replacement and migration of ILCs into the intestine and their specific effects on ILC3 subsets will need to be addressed in future studies.

Herein, we highlighted the relevance of hypoxia-induced HIF-1α stabilization in ILC3 and its consequence to other cells in the intestinal mucosa, such as ILC1 and IECs. Our findings also indicate new therapeutic possibilities, such as drugs that regulate HIF-1α stability, which could be applied to patients with chronic intestinal inflammation or enteric infections.

## Methods

### Mice

Adult C57BL/6 male mice were purchased from the Multidisciplinary Centre for Biological Investigation, Campinas-SP, Brazil. Rorc-Cre, Villin-Cre, and HIF-1alfa^floxed/floxed^ mice were from The Jackson Laboratories. All strains were maintained in a C57BL/6 background and kept in regular filter-top cages with free access to sterile water and food. Animal procedures were approved by the Ethics Committee on Animal Use of the University of Campinas (protocol #5495–1/2020).

### Intestinal innate lymphoid cell isolation

Small intestinal or colon samples were harvested, and mesenteric adipose tissue and Peyer’s patches removed by dissection before cutting longitudinally and washing out the contents. Intraepithelial lymphocytes were excluded by two 20 min extraction washes with 5 mM EDTA. Intestinal lamina propria immune cells were isolated by digestion with 1 mg/mL collagenase IV (Sigma) for 40 min at 37 °C and shaking. Lymphocytes were enriched over a Percoll gradient (GE Healthcare) (40%/70% interface). Cells were washed, counted in Neubauer’s chamber and stimulated ex vivo and/or labeled with monoclonal antibodies for analysis by flow cytometry (Table S1).

### Cell culture

The MNK3 cell line was previously described as an in vitro system to study ILC3 functionality.^46^ MNK3 cells or single-cell preparations of small intestine lamina propria were cultured in complete RPMI (Corning), containing 10% fetal bovine serum, 2 mM GlutaMAX, 1 mM sodium pyruvate, 55 μM 2-mercaptoethanol, 50 μg/mL gentamicin and 10 mM HEPES (ThermoFisher). Conditioned medium containing IL-2 and IL-7 was also used to maintain cells. For hypoxia assays, 2 × 10^5^ cells were cultured in polystyrene plates in 96 round-bottomed wells (Corning). Plates were placed into a horizontal sealed jar with an anaerobic gas generator (AnaeroGen, Oxoid; ThermoFisher Scientific), which reduces the oxygen level to <1% within 3 h. Cells were kept in hypoxic atmosphere for 3 h at 37 °C and viability was assessed using the FITC Annexin V Apoptosis Detection Kit (BD Biosciense). Normoxic controls were incubated during the same period under 21% O_2_ and 5% CO_2_ at 37 °C. Additional experiments were performed under controlled 8% O_2_ levels. Cells were in the Cytation 5 Multi‐Mode Reader equipped with a Gas controller (BioTek Instruments). For in vitro assays, cells were cultured in complete RPMI with or without a combination of IL-1β (10 ng/mL) and IL-23 (10 ng/mL) in the presence or absence of Bay 87–2243/HIF-1 inhibitor (1–100 µM, Sigma-Aldrich), Bay 85–3934/HIF-1α stabilizer (1–100 µM, Sigma-Aldrich), rapamycin (20 nM; Millipore), deoxyglucose (2DG, 1 mM, Sigma-Aldrich) and brefeldin A (1:1000, BD Golgi Plug), for 3 h under hypoxia or normoxia.

### Flow cytometry

To identify ILC populations, dead single-cell preparations of small intestine or colon lamina propria were excluded using a live/dead cell viability assay in Brilliant Violet 510. A lineage cocktail containing R-phycoerythrin-conjugated monoclonal antibodies against CD3, CD5, CD19, CD11c, and CD11b was used except where stated and population were identified as Lin^−^ and CD45^+^ (PE-Cy7), as already reported^19–21^ (Supplementary Fig. S1B). Surface staining was performed with antibodies diluted in FACS-buffer at 4 °C for 20 min in the dark after blockade of Fc receptors with purified anti-CD16/CD32 (Biolegend). Cells were fixed and intracellularly stained using the Foxp3 Staining Buffer Set (eBioscience) according to manufactures instructions, using monoclonal antibodies to RORγt (Percp-Cy5.5), Gata3 (FITC), and T-bet (APC). For functional experiments, MNK3 and small intestine lamina propria (si-LP) cells were cultured in 96-well plates in complete media and stimulated in the presence of Golgi Plug (BD Bioscience) for 3 h at 37 °C. Following incubation, cells were stained for viability and surface molecules, fixed with 2% paraformaldehyde (PFA) and intracellular staining performed using BD Biosciences Fixation/Permeabilization Solution Kit or eBioscience Transcription Factor staining Kit for cytoplasmatic or nuclear proteins, such as cytokines and transcription factors (Supplementary Table S1). The ILC3-enriched cell population was identified as Live^+^ Lin^–^ (CD3^–^CD5^–^CD19^–^CD11b^–^CD11c^–^) CD45^low^CD90.2^high^ (Supplementary Fig. S1A), as already reported.^19–21^ For the proliferation assay, cells were incubated in complete RPMI with EdU or DMSO (20 µM, Thermo-Fisher Scientific, MA, USA) for 3 h at 37 °C, under hypoxia/normoxia. Following incubation, cells were fixed in 4% PFA and permeabilized with PBS containing 0.05% triton X-100. Detection of EdU-DNA was performed using the Click-iT™ EdU Cell Proliferation Kit, Alexa Fluor™ 647 dye (Thermo-Fisher Scientific). Ki67 was stained with a monoclonal antibody after fixing/permeabilizing in vitro cultured cells. For glucose uptake, using 2-deoxy-2-[(7-nitro-2,1,3-benzoxadiazol-4-yl)amino]-D-glucose (2-NBDG, 10 µg/mL; Invitrogen), cells were incubated in RPMI medium for 2 h at 37 °C under normoxia and hypoxia and measured by flow cytometry. Samples were analyzed on BD FACS-Verse™ (BD Biosciences) using BD FACSuite™ Software (BD biosciences). All FACS data were analyzed using FlowJo v.9.5.2 software (Tree Star).

### Quantitative gene expression

Total RNA was extracted from tissue using the PureLink^TM^ RNA kit (Ambion). RNA was converted to cDNA using the High-Capacity cDNA Reverse Transcription Kit (Applied Biosystems) and qPCR was performed using Power SYBR Green PCR Master Mix (Applied Biosystems) and primers indicated in Supplementary Table S2. Quantification of gene expression was performed using 2^ΔΔ^Ct method with β2-microglobulin as a reference gene.

### Hypoxia chamber

For acute hypoxic exposures, mice were housed in a normobaric gas chamber (Biospherix, Parish, NY) with circadian cycle/air humidity control, containing continuous electronic nitrogen injection. Oxygen concentrations were maintained at 8–9%, as described by Rempel et al.^47^ After 48 h, mice under hypoxia and their littermate controls under normoxia (21% O_2_) were euthanized and tissues harvested to analyze the ILC3 activation.

### *C. difficile* infection

The *C. difficile* VPI 10463 strain was cultivated in BHI blood agar supplemented with hemin (5 μg/mL) and menadione (1 μg/mL) at 37 °C in anaerobic atmosphere (AnaeroGen, Oxoid; ThermoFisher Scientific) in jars. 8–10-wk-old age- and gender-matched mice were infected as previously described.^48^ Mice were pre-treated with antibiotic mixture (0.4 mg/mL kanamycin, 0.035 mg/mL gentamicin, 0.035 mg/mL colistin, 0.215 mg/mL metronidazole, and 0.045 mg/mL vancomycin; Sigma) added to drinking water for 4 days. Next, antibiotics were discontinued and mice received an intraperitoneal single dose of clindamycin (10 mg/kg) (Sigma). After 1 day, mice were infected with 1 × 10^8^ colony forming units (CFUs) *C. difficile* by gavage. Mice were weighed and monitored daily for clinical severity scores that varied from 0 (normal) to 15 (dead) (Supplementary Table S3).

### Histological analysis

Mouse colons were harvested, opened longitudinally and fixed in 4% formalin/0.1% glutaraldehyde. Tissues were processed into histo-resin and 5-µm sections prepared for staining with Hematoxylin and Eosin solution. Slides were photographed and analyzed using a U-LH100HG Olympus Microscope with 20× objective lens.

### Bacterial translocation

Spleen, liver and mesenteric lymph nodes were harvested on day 5 of *C. difficile* infection. Bacterial 16S rDNA was extracted using the PureLink^TM^ Microbiome DNA Purification kit (ThermoFisher Scientific) and gene levels were quantified by qPCR using primers complementary to Eubacteria 16S rDNA conserved region (Supplementary Table S2). Bacterial load was determined using an *E. coli* genomic DNA standard curve and the CFU/g tissue was normalized (gene levels/sample weight).

### Lactate measurement

MNK3 cells were cultured in 96-well plates at 2 × 10^5^ cells per well in complete RPMI medium under hypoxic or normoxic atmosphere for 3 h. Cells were stimulated or not with a combination of IL-1β (10 ng/mL), IL-23 (10 ng/mL) and rapamycin (20 nM). Following incubation, the supernatant was collected and used for measurement of lactate release with an Enzymatic Lactate Kit, according to manufacturer’s instructions (Labtest, MG—Brazil). The absorbance was read at 530 nm and lactate concentrations (mg/dL) determined using a standard control provided in the kit.

### Mitochondrial mass

MNK3 cells were seeded in triplicate in 96-wells plate at 2 × 10^5^ cells/well in complete RPMI (containing 4% IL7 and 2% IL2) and cultured under hypoxic/normoxic atmosphere for 3 h at 37 °C. MitoTracker Green (for total mitochondrial mass) and MitoTracker Red (for mitochondrial membrane potential) staining were performed according to manufacturer’s instructions (Invitrogen). Dysfunctional mitochondria were measured as described.^49^ Data were acquired with BD FACS-Verse™ flow cytometer (BD Biosciences) and analyzed with FlowJo software (Tree Star).

### Seahorse metabolic analysis

Real-time analysis of extracellular acidification rate (ECAR) and OCR was performed using an XFe24 Analyzer (Seahorse Bioscience). MNK3 cells were seeded 2 h in quadruplicate at 1 × 10^6^ cells per well on a pretreated poly-D-lysine-coated 24-well Seahorse plate in complete RPMI containing 4% IL7 and 2% IL2. Cells were washed and cultured in Seahorse Assay Medium containing 10 mM glucose and 1 mM sodium pyruvate for 2 h at 37 °C without CO_2_. Oligomycin (ATPase inhibitor, 1 μM), FCCP (uncoupler of mitochondrial oxidative phosphorylation, 1 μM), rotenone (complex I inhibitor, 1 μM) and antimycin A (complex III inhibitor, 1 μM) were injected where indicated and the ECAR (mpH/min) and OCR (pmol O_2_/min) were measured in real time.

### Chromatin immunoprecipitation (ChIP)

MNK3 cells were cultured in 12-well plates at 2 × 10^6^ cells/well in complete RPMI medium under hypoxic or normoxic atmosphere for 3 h at 37 °C. Subsequently, cells were fixed in 1% formaldehyde in PBS for 10 min, quenched with 0.1 M glycine and processed for ChIP using the MAGnify^TM^ Chromatin Immunoprecipitation System (Invitrogen), following the manufacturer’s instructions. ChIP reactions were performed using 3 µg anti-HIF-1α antibody/sample (Supplementary Table S1). Proteins complexes were reverse crosslinked at 65 °C overnight and digested with proteinase K (0.2 µg/µL) for 1 hour at 55 °C. Samples were purified using the Qiagen DNA Purification Kit according to the manufacturer’s instructions. Purified DNA was used for qPCR analysis. Input samples (total chromatin extraction, before immunoprecipitation) were analyzed in parallel and used to calculate enrichment in the precipitate by log_2_-fold change. Primer sequences are listed in Supplementary Table S2.

### Statistical analysis

Analyses were performed using GraphPad software 8.0 (San Diego, CA, USA). All data are presented as mean ± SEM. All experiments were repeated at least twice, except the Seahorse and hypoxic chamber in vivo exposure assays. Differences were considered significant for *p* < 0.05. Results were compared by Student’s *t* test or Mann Whitney test, as appropriate. For more than two groups, differences were compared by one-way analysis of variance (ANOVA) followed by Tukey’s post hoc test.

## Supplementary information

SUPPLEMENTARY MATERIAL
