# Correction to: Hypoxia enhances ILC3 responses through HIF-1α-dependent mechanism

**DOI:** 10.1038/s41385-021-00403-9

**Published:** 2021-04-16

**Authors:** J. L. Fachi, L. P. Pral, J. A. C. dos Santos, A. C. Codo, S. de Oliveira, J. S. Felipe, F. F. F. Zambom, N. O. S. Câmara, P. M. M. M. Vieira, M. Colonna, M. A. R. Vinolo

**Affiliations:** 1 Laboratory of Immunoinflammation, Department of Genetics, Evolution, Microbiology and Immunology, Institute of Biology, University of Campinas, Campinas, Brazil; 2 Department of Pathology and Immunology, Washington University School of Medicine, Saint Louis, MO USA; 3 Laboratory of Immunometabolism, Department of Genetics and Evolution, Microbiology and Immunology, Institute of Biology, University of Campinas, Campinas, Brazil; 4 Renal Division, Department of Clinical Medicine, Faculty of Medicine, University of São Paulo, São Paulo, Brazil; 5 Department of Immunology, Institute of Biomedical Sciences, University of São Paulo, São Paulo, Brazil; 6 Experimental Medicine Research Cluster, Campinas, Brazil; 7 Obesity and Comorbolities Research Center (OCRC), University of Campinas, Campinas, Brazil

Correction to: *Mucosal Immunology* 10.1038/s41385-020-00371-6, published online 14 January 2021

The article Hypoxia enhances ILC3 responses through HIF-1α-dependent mechanism, written by J. L. Fachi, L. P. Pral, J. A. C. dos Santos, A. C. Codo, S. de Oliveira, J. S. Felipe, F. F. F. Zambom, N. O. S. Câmara, P. M. M. M. Vieira, M. Colonna and M. A. R. Vinolo, was originally published electronically on the publisher’s internet portal on 14 January 2021 without open access. With the author(s)’ decision to opt for Open Choice the copyright of the article changed on 18 March 2021 to © The Author(s) 2021 and the article is forthwith distributed under a Creative Commons Attribution 4.0 International License, which permits use, sharing, adaptation, distribution and reproduction in any medium or format, as long as you give appropriate credit to the original author(s) and the source, provide a link to the Creative Commons licence, and indicate if changes were made.

The images or other third party material in this article are included in the article’s Creative Commons licence, unless indicated otherwise in a credit line to the material. If material is not included in the article’s Creative Commons licence and your intended use is not permitted by statutory regulation or exceeds the permitted use, you will need to obtain permission directly from the copyright holder.

To view a copy of this licence, visit http://creativecommons.org/licenses/by/4.0/

