# Supplementary material for: Hypoxia enhances ILC3 responses through HIF-1α-dependent mechanism
Source: Mucosal Immunol. 2021 Jan 14;14(4):828–41. doi: 10.1038/s41385-020-00371-6 (PMC8221997; doi:10.1038/s41385-020-00371-6)
Supplement: Supplementary file 1 — SUPPLEMENTARY MATERIAL [file 41385_2020_371_MOESM1_ESM.docx]

**SUPPLEMENTARY MATERIAL**


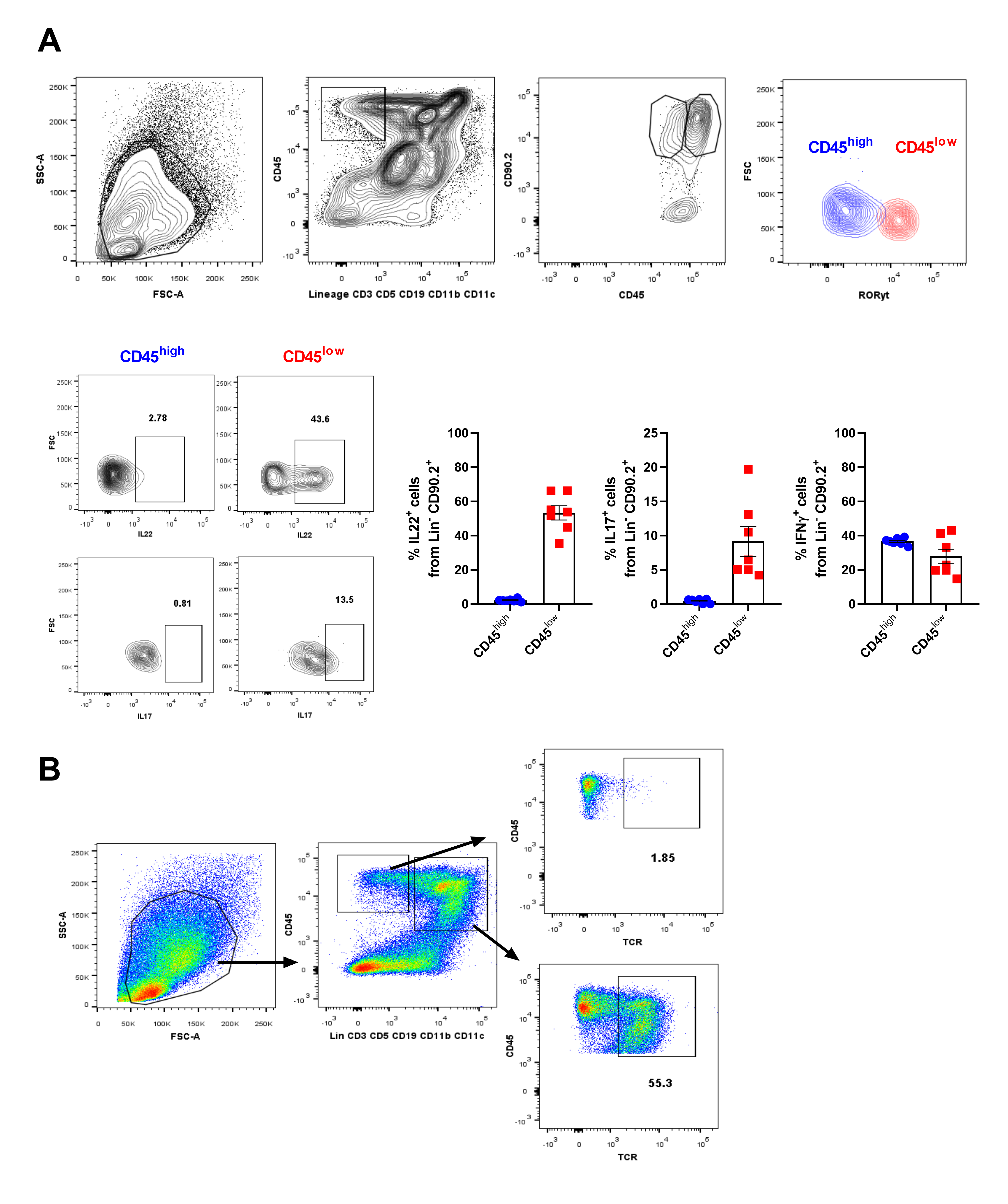


**Fig. S1 - Gating strategy for identification of ILC3-enriched population.**

**(A)** Gating strategy used for ILC identification from small intestine (si) lamina propria. Cells were gated on lineage negative (CD3, CD5, CD19, CD11c, and CD11b) and CD45 positive. CD90.2 positive cells were divided in two subpopulations: Lin^-^CD45^low^CD90.2^+^ and Lin^-^CD45^high^CD90.2^+^, indicated as CD45^low^ and CD45^high^, respectively. The enriched ILC3 population (CD45^low^) expressed higher levels of RORγt and IL-22/IL-17 cytokines than the CD45^high^ population. Results are presented as mean ± SEM. (**B**) Identification of T cell contamination in the gates. Cells were gated on lineage negative (CD3, CD5, CD19, CD11c, and CD11b) and CD45 positive. T cell contamination was analyzed by TCR staining.


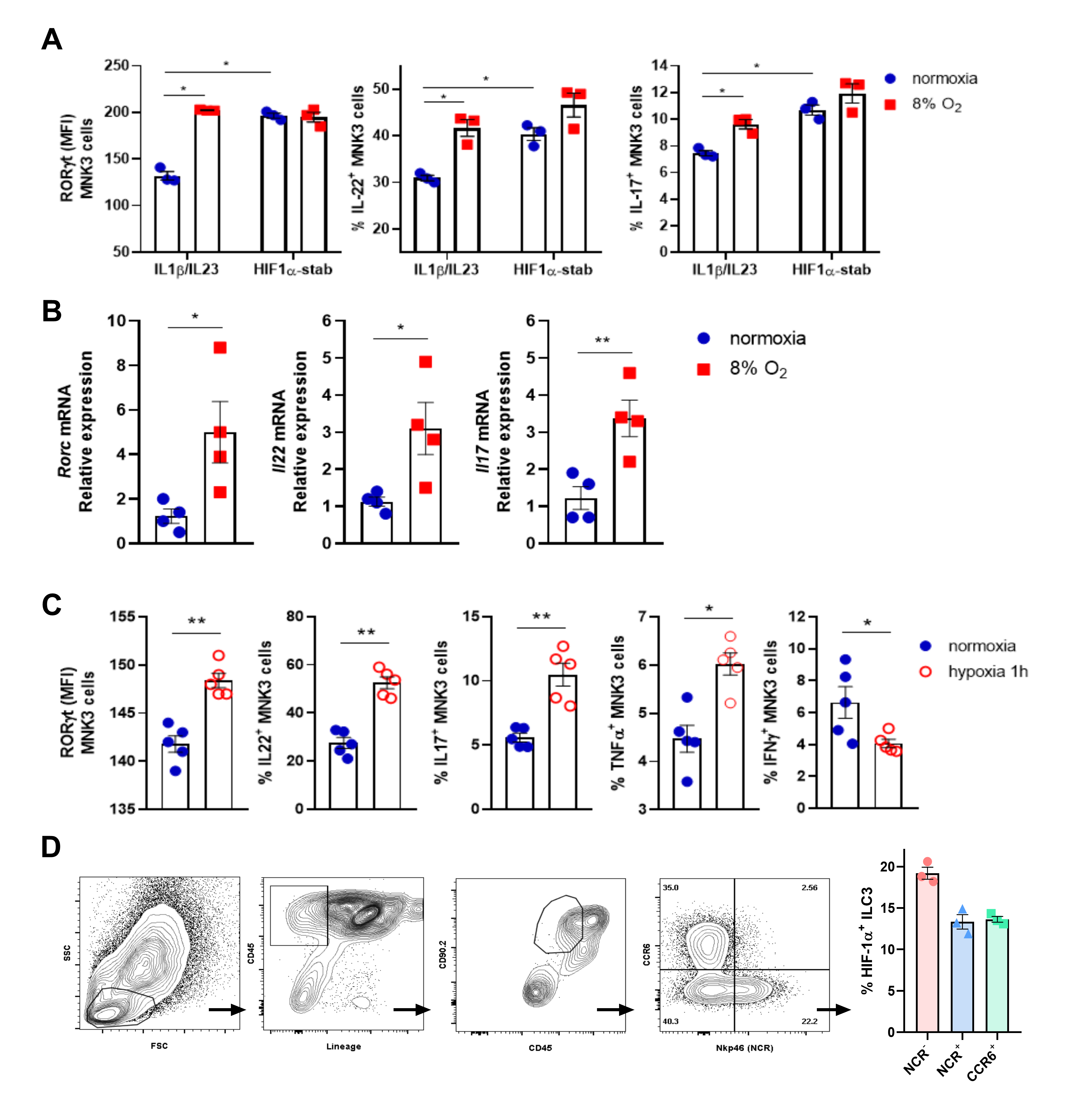


**Fig. S2 - Hypoxia induces ILC3 activation and HIF-1α expression.**

**(A)** RORγt MFI and percentage of IL17 and IL22 producing MNK3 cells, stimulated with IL-1β + IL-23 and treated or not with a HIF-1α stabilizer during 3 hours under normoxia or 8% O_2_ (n = 3). **(B)** Relative *Rorc*, *Il22* and *Il17* mRNA expression by stimulated MNK3 under normoxia or 8% O_2_-hypoxia (n = 4). **(C)** RORγt MFI and IL22, IL17, TNFα, and IFNγ production by MNK3 cells stimulated during 1 hour in normoxia or hypoxia (n = 5). (**D**) HIF-1α content was assessed in ILC3s subsets by flow cytometry. Cells were stimulated with IL1β/IL23 for 3 hours under hypoxia and then analyzed for HIF-1a content. Nkp46 (NCR)^+^, NCR^-^ and CCR6^+^ cells were analyzed in ILC3-enriched cell population (n = 3). Gating strategy is on the left. Results are presented as mean ± SEM. * p<0.05; **p<0.01.


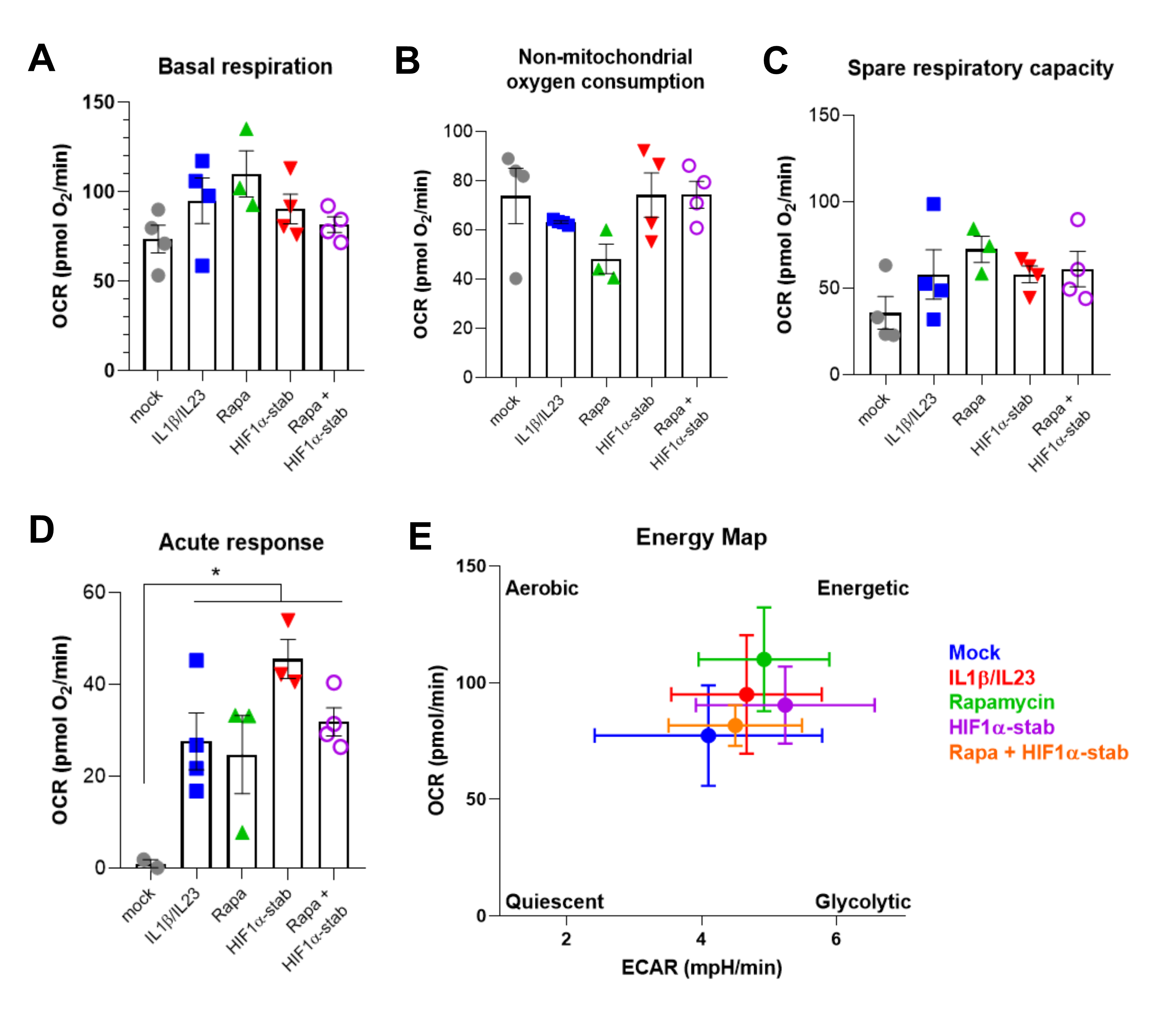


**Fig. S3 - HIF1 signaling does not rely on mitochondrial respiration.**

**(A, E)** MNK3 cells basal respiration (A), non-mitochondrial oxygen consumption (B), spare respiratory capacity (C), acute response (D) in OCR, and energy map in ECAR vs OCR (E) after stimulation and treatment with oligomycin (OLF), FCCP, and rotenone/antimycin A (R/A) (n = 4). Cells were stimulated or not with IL-1β + IL-23 and treated or not with a HIF-1α stabilizer and/or rapamycin. Results are presented as mean ± SEM. * p<0.05.





**Fig. S4 – IEC-derived cytokines do not seem to explain the ILC3 phenotype observed in hypoxia or *in vivo*.**

**(A, B)** Relative *Il23r* and *Il1r* mRNA expression by MNK3 under normoxia or hypoxia (A) and primary si-ILC3 from HIF1α^ΔRorc^ and HIF1α^floxed^ mice (n = 4). **(C, D)** Small intestine length (C) and absolute number of Lin^-^CD45^+^ ILC subsets (D) from small intestine lamina propria of HIF1α^ΔIEC^ and HIF1α^floxed^ mice at steady state (n = 4-5). **(E, G)** Transcription factors MFI in Lin^-^CD45^+^ ILCs (E), Percentage of Ki67^+^ ILC3 (F), and percentage of producing IL22, IL17 and IFNγ ILC3 from small intestine lamina propria of HIF1α^ΔIEC^ and HIF1α^floxed^ mice (n = 4-5). Results are representative of at least two independent experiments and Results are presented as mean ± SEM. * p<0.05; **p<0.01.


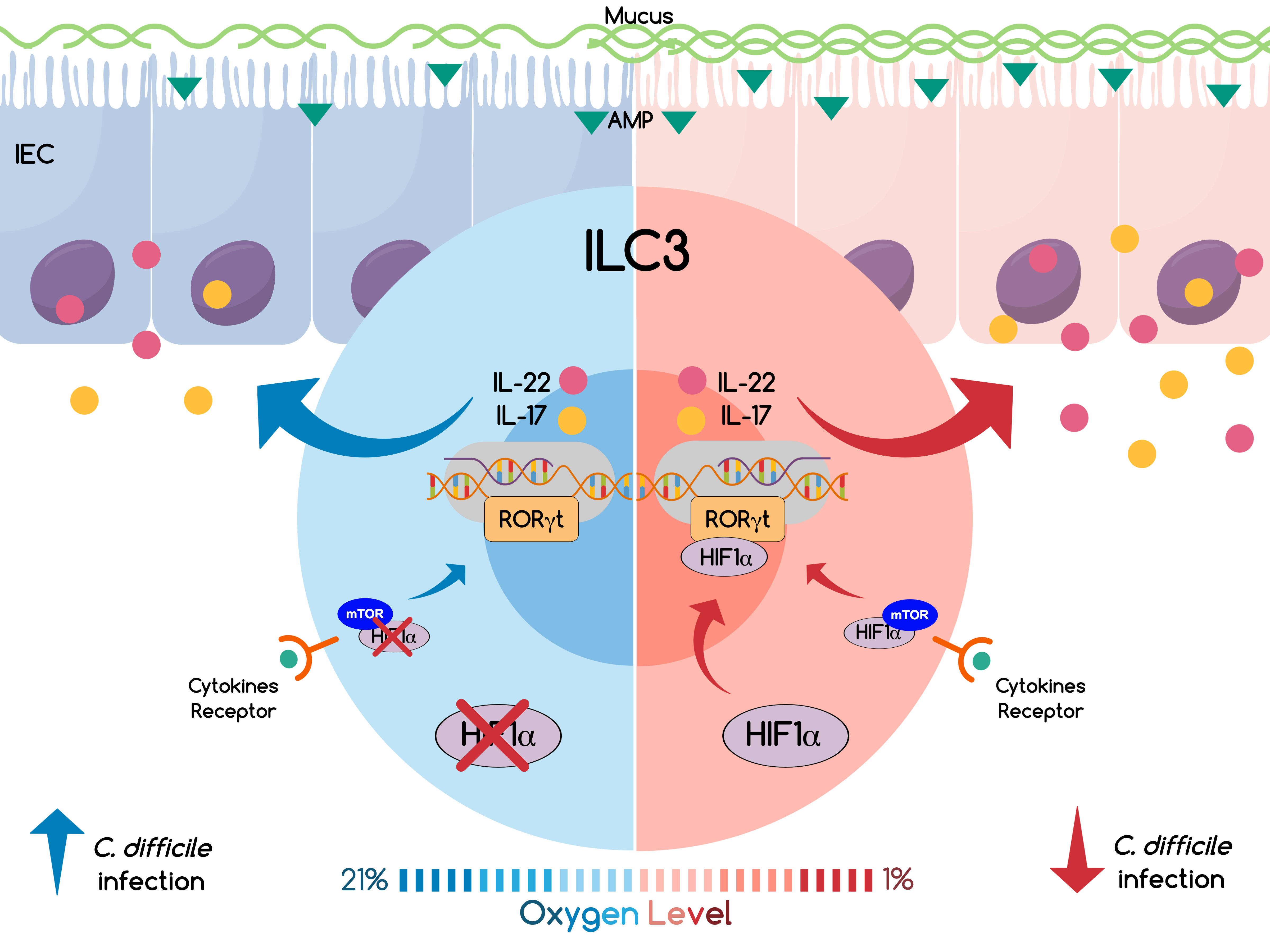


**Fig. S5 - Graphical abstract.**

Oxygen fluctuation supports the intestinal ILC3 responses through a HIF-1α-dependent phenotype. Low O_2_ levels and cytokine stimulation, such as IL1β and IL23, increase the intracellular HIF-1α accumulation and its transcriptional fate, improving the expression of RORγt and, consequently, IL17 and IL22 production. The silencing of HIF-1α, through pharmacological inhibitors of mTORC1-HIF-1α or gene knockout, validates its direct ability to modulate ILC3 responses under hypoxia. Overactivated ILC3, from its cytokines, stimulates the functionalities of intestinal epithelial cells, such as mucus production and antimicrobial peptides, and deals with intestinal homeostasis. Likewise, the absence of HIF-1α signaling increases the host's susceptibility to intestinal infection by *Clostridioides difficile*, exhibiting greater tissue impairment and loss of the ILC3-mediated protective immune response.

**Table S1.** Antibodies.

| **ANTIBODY** | **SOURCE** | **CLONE** | **IDENTIFIER** |
| --- | --- | --- | --- |
| Anti-mouse CD45, PE/Cy7 | eBioscience™ | 30-F11 | Cat# 25-0451-82 |
| Anti-mouse CD3, PE | BioLegend | 17A2 | Cat# 100206 |
| Anti-mouse CD5, PE | BioLegend | 53-7.3 | Cat# 100608 |
| Anti-mouse CD19, PE | BioLegend | 6D5 | Cat# 115508 |
| Anti-mouse CD11b, PE | BioLegend | M1/70 | Cat# 101208 |
| Anti-mouse CD11c, PE | BioLegend | N418 | Cat# 117308 |
| Anti-mouse Ly6G, PE | BioLegend | 1A8 | Cat# 127608 |
| Anti-mouse CD45 APC/Cy7 | BioLegend | 30-F11 | Cat# 103116 |
| Anti-mouse CD90.2 (Thy-1.2) FITC | BioLegend | 53-2.1 | Cat# 140303 |
| Anti-mouse CD90.2 (Thy-1.2) Brilliant Violet 785™ | BioLegend | 30-H12 | Cat# 105331 |
| Anti-mouse CD335 (NKp46), Brilliant Violet 421™ | BioLegend | 29A1.4 | Cat# 137612 |
| Anti-mouse CD196 (CCR5), PerCP/Cy5.5 | BioLegend | 29-2L17 | Cat# 129809 |
| Anti-mouse RORγt, PerCP/Cy5.5 | BD Pharmingen™ | Q31-378 | Cat# 562683 |
| Anti-mouse GATA3, Alexa Fluor-488 | BioLegend | 16E10A23 | Cat# 653808 |
| Anti-mouse Tbet, APC | Biolegend | 4B10 | Cat# 644814 |
| Anti-mouse IL17, APC-Cy7 | BD Pharmingen™ | TC11-18H10 | Cat# 560821 |
| Anti-mouse IL22 APC | BioLegend | Poly5164 | Cat# 516409 |
| Anti-mouse IFNy, Brilliant Violet 421™ | BioLegend | XMG1.2 | Cat# 505830 |
| Anti-mouse TCR β-chain, APC | BioLegend | H57-597 | Cat# 109212 |
| Anti-TNFα, PE | BioLegend | MP6-XT22 | Cat# 506306 |
| Anti-human/mouse HIF-1α, Alexa Fluor® 488 | R&D System | 241812 | Cat# IC1935G |
| Anti-human/mouse HIF-1α | Abcam | mgc3 | Cat# ab16066 |
| Anti-mouse IgG1, FITC | eBioscience™ | M1-14D12 | Ca# 11-4015-82 |
| Anti-mouse Ki67, PerCP/Cy5.5 | BD Pharmingen™ | B56 (RUO) | Cat# 561284 |
| FITC Annexin-V Apoptosis Detection Kit with 7-AAD | BioLegend | - | Cat# 640922 |
| Live/Dead™ Fixable Aqua Dead Cell Stain Kit, BV510 | Invitrogen™ | - | Cat# L34957 |

**Table S2.** qPCR primers.

| **Primer** | | **Sequence (5’ – 3’)** |
| --- | --- | --- |
| ***Eubacteria* (rDNA 16S)** | Forward | ACT CCT ACG GGA GGC AGC AGT |
|  | Reverse | ATT ACC GCG GCT GCT GGC |
| ***B2m*** | Forward | CCC CAC TGA GAC TGA TAC ATA CG |
|  | Reverse | CGA TCC CAG TAG ACG GTC TTG |
| ***Il17*** | Forward | TCA GCG TGT CCA AAC ACT GAG |
|  | Reverse | GAC TTT GAG GTT GAC CTT CAC AT |
| ***Il22*** | Forward | AGA ATG TCA GAA GGC TGA AGG |
|  | Reverse | AGG AGC AGT TCT TCG TTT TCT AG |
| ***Rorc*** | Forward | TCC ACT ACG GGG TTA TCA CCT |
|  | Reverse | AGT AGG CCA CAT TAC ACT GCT |
| ***Ldha*** | Forward | ACG CAG ACA AGG AGC AGT GGA A |
|  | Reverse | ATG CTC TCA GCC AAG TCT GCC A |
| ***Slc2a1*** | Forward | CTT TGT GGC CTT CTT TGA AGT |
|  | Reverse | CCA CAC AGT TGC TCC ACA T |
| ***Pfkfb3*** | Forward | GGA GGT CGG CAT GTT GAA GA |
|  | Reverse | CTT TGG AAG GGC CTG AGA GG |
| ***Tff3*** | Forward | TGC AGA TTA CGT TGG CCT GT |
|  | Reverse | TGC AGA GGT TTG AAG CAC CA |
| ***Hif1a*** | Forward | ATC TCG GCG AAG CAA AGA GTC |
|  | Reverse | TGG GGA AGT GGC AAC TGA T |
| ***Il1r*** | Forward | GCA CGC CCA GGA GAA TAT GA |
|  | Reverse | AGA GGA CAC TTG CGA ATA TCA A |
| ***Il23r*** | Forward | GAG GAC ATC CTG CTT CAG GTA AT |
|  | Reverse | AGC CAC TTT GGG ATC ATC AGT A |
| ***Tbx21*** | Forward | AAC CGC TTA TAT GTC CAC CCA |
|  | Reverse | CTT GTT GTT GGT GAG CTT TAG C |
| ***Gata3*** | Forward | GCG GGC TCT ATC ACA AAA TGA |
|  | Reverse | GCT CTC CTG GCT GCA GAC AGC |
| ***Ahr*** | Forward | GCC CTT CCC GCA AGA TGT TAT |
|  | Reverse | GCT GAC GCT GAG CCT AAG AAC |
| ***Bcl2*** | Forward | TGA GTA CCT GAA CCG GCA TCT |
|  | Reverse | GCA TCC CAG CCT CCG TTA T |
| ***Ccnd1*** | Forward | GCA AGC ATG CAC AGA CCT T |
|  | Reverse | GTT GTG CGG TAG CAG GAG A |
| ***Ocln*** | Forward | CTC TCA GCC AGC GTA CTC TT |
|  | Reverse | CTC CAT AGC CAC CTC CGT AG |
| ***Cldn1*** | Forward | CACTCCCAGACTCCACCACC |
|  | Reverse | CGATCCATCCCAGAGAAGCC |
| ***Muc1*** | Forward | CCC TAC CTA CCA CAC TCA CGG ACG |
|  | Reverse | GTG GTC ACC ACA GCT GGG TTG GT |
| ***Muc2*** | Forward | CGA CTG TGA GCA GTG TGT CA |
|  | Reverse | GGG TAG GGT CAC CTC CAT CT |
| ***Muc4*** | Forward | GAG GGC TAC TGT CAC AAT GGA GGC |
|  | Reverse | AGG GTT CCG AAG AGG ATC CCG TAG |
| ***Reg3g*** | Forward | TTC CTG TCC TCC ATG ATC AAA A |
|  | Reverse | CAT CCA CCT CTG TTG GGT TCA |
| ***Defb1*** | Forward | CCA GAT GGA GCC AGG TGT TG |
|  | Reverse | CTG GAG CGG AGA CAG AAT CC |
| ***Defb3*** | Forward | GCA TTG GCA ACA CTC GTC AGA |
|  | Reverse | CGG GAT CTT GGT CTT CTC TA |
| ***Camp*** | Forward | TCTCTACCGTCTCCTGGACCTG |
|  | Reverse | CCACATACAGTCTCCTTCACT |
| ***Cxcl1*** | Forward | ACT GCA CCC AAA CCG AAG TC |
|  | Reverse | TGG GGA CAC CTT TTA GCA TCT T |
| ***Cxcl2*** | Forward | GGG ACA AAT AGC TGC AGT CGG |
|  | Reverse | CTA CTC TCC TCG GTG CTT AC |
| ***Il1b*** | Forward | GGC AGC TAC CTG TGT CTT TCC C |
|  | Reverse | ATA TGG GTC CGA CAG CAC GAG |
| ***Il16*** | Forward | CTG CAA GAG ACT TCC ATC CAG |
|  | Reverse | AGT GGT ATA GAC AGG TCT GTT GG |
| ***Il2*** | Forward | CCT GAG CAG GGA GAA TTA CA |
|  | Reverse | TCC AGA ACA TGC CGC AGA |
| ***Csf2*** | Forward | ACC ACC TAT GCG GAT TTC AT |
|  | Reverse | TCA TTA CGC AGG CAC AAA AG |
| ***Tnfa*** | Forward | TCT TCT CAT TCC TGC TTG TGG C |
|  | Reverse | CAC TGG TGG TTT GCT ACG ACG |
| ***Ifng*** | Forward | ATG AAC GCA CAC ACT GCA TC |
|  | Reverse | CCA TCC TTT TGC CAG TTC CTC |
| ***Serpine1* (promoter)** | Forward | AGC CCC ACC CAC TTT CTA AC |
|  | Reverse | TTA TCC CCC ATG CCC TTT CAC |
| ***Aldoa* (promoter)** | Forward | CGA AAC AGA FGC AGT TGA GG |
|  | Reverse | TTA GCC CCT TTG CTT ACC C |
| ***Lox* (promoter)** | Forward | CTC CCT GTG CAA CGT GTC T |
|  | Reverse | TGC AGT TAC ACA AGC CGT TC |
| ***Rorc* (promoter; HIF-1 binding site)** | Forward | GAA TTT TCC AAC GCC CCC TG |
|  | Reverse | CTC TGC TCC ACA CAG GTG GC |
| ***Rorc* (promoter; non-HIF-1 binding site)** | Forward | CAA TCC TCC GTG CTG ACA GCA |
|  | Reverse | CTG TCT AAG GGC GAA GGT CA |
| ***Il17f* (promoter; RORγt flanking site)** | Forward | GTC TAT CGT GTA TCT GAC CCG |
|  | Reverse | CCA ATG CTG AGA TTA AAG GC |
| ***Il17f* (promoter; non-RORγt flanking site)** | Forward | AGG TGG ATA TCT GTA CAT TAG |
|  | Reverse | TCT ATA CAT TGT CCC ATT GTG |
| ***Il22* (promoter)** | Forward | TGG TGG GAA AAT GAG TCC GT |
|  | Reverse | AGA GGT CTG AGT GCT TAC CTG |
| ***Dnajb6* (promoter)** | Forward | TGG ATT CAG CAC TGC GAG TT |
|  | Reverse | GGT TCC TGC ACC CGT ATT CC |
| ***Egln3* (promoter)** | Forward | AGG AAC GAG AGA AAG CAC CG |
|  | Reverse | GAA CAG CCA GAC CCG AAT CA |

**Table S3.** Clinical score.

| **Category** | **Score*** |  |  |  |
| --- | --- | --- | --- | --- |
|  | **0** | **1** | **2** | **3** |
| **Activity** | Normal | Alert/Slow moving | Lethargic/Shaky | Inactive unless prodded |
| **Posture** | Normal | Back slanted | Hunched | Hunched/Nose down |
| **Coat** | Normal | Piloerection | Rough skin | Very ruffled Puff/Ungroomed |
| **Diarrhoea** | Normal | Soft stool/Discoloured (yellowish) | Wet stained tail/ mucous +/- blood | Liquid/no stool (ileus) |
| **Eyes/Nose** | Normal | Squinted  ^1^/_2_ closed | Squinted/Discharge | Closed/Discharge |

*Clinical score = sum of all parameters scores. Total possible score is 15 (death).
